# Computational Fluid Dynamic Model Prediction of Enhanced Glymphatic Clearance in Response to Focused Ultrasound‐Mediated Blood‐Brain Barrier Opening

**DOI:** 10.1002/advs.202510684

**Published:** 2025-09-04

**Authors:** Ryan A. Gladwell, Delaney G. Fisher, Joshua D. Wythe, Christopher B. Highley, John R. Lukens, Richard J. Price

**Affiliations:** ^1^ Department of Biomedical Engineering University of Virginia Charlottesville VA 22908 USA; ^2^ Department of Cell Biology University of Virginia Charlottesville VA 22908 USA; ^3^ Department of Neuroscience University of Virginia Charlottesville VA 22908 USA

**Keywords:** Alzheimer's Disease, blood‐brain barrier, computational modeling, focused ultrasound, glymphatic system, Parkinson's Disease

## Abstract

Focused Ultrasound (FUS) is the concentration of acoustic energy into a small region to produce therapeutic bioeffects. FUS‐induced blood‐brain barrier opening (BBBO), a strategy to deliver drugs and genes to the brain, also enhances glymphatic drainage, the brain‐specific waste clearance system. Thus, FUS BBBO is a promising strategy for addressing the accumulation of neurotoxic solutes that are characteristic of many neurodegenerative diseases. However, the biotransport mechanisms by which FUS augments glymphatic drainage are not well understood. To address this knowledge gap, a 3D finite element COMSOL model of a single penetrating arteriole‐venule vascular unit in the brain is engineered. The model predicts that i) FUS greatly improves waste clearance by increasing both diffusion and convection, ii) the convection‐mediated movement of cerebrospinal fluid (CSF) and solute from the arteriole to the venule is largely dependent on the diffusion‐mediated interactions between the solute, the medium through which it is moving, and CSF and iii) solutes more centralized to the CSF flow profile and that have a higher diffusion coefficient tend to clear more rapidly due to increased convection and enhanced diffusive mixing. The computational model can both inform therapeutic strategies and elucidate mechanisms of secondary responses to FUS BBBO.

## Graphical abstract

Focused ultrasound‐mediated blood‐brain barrier opening (FUS BBBO) for enhanced drug delivery also augments glymphatic clearance. A computational model of an arteriole‐venule pair in the brain offers insight into the underlying biotransport mechanisms responsible for this mass clearance effect. Flux is largely dependent on the implicit interactions between the solute and the medium through which it is moving.

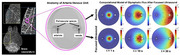

## 1 Introduction

Any energetic cellular process generates by‐products that, if not disposed of, can disrupt cellular, organ, and organismal homeostasis. In the metabolically active brain, protein accumulation is a hallmark etiology of many neurodegenerative disorders, such as the build‐up of amyloid‐β‐ β in Alzheimer's Disease (AD) or ɑ‐synuclein in Parkinson's Disease.^[^ ^1^ ^]^ Whereas homeostasis in peripheral tissues is maintained via fluid and waste clearance by the lymphatic vasculature and circulating immune cells, respectively, the mammalian brain has evolved two systems to actively clear soluble proteins and metabolic waste and regulate immune cell trafficking: active surveillance and phagocytosis by residential glial cells and passive maintenance through the glymphatic (glial‐dependent lymphatic) system.^[^ ^2^, ^3^, ^4^ ^]^

Despite their functional connectivity, the glymphatic system differs significantly from peripheral lymphatics. Indeed, no vessels in the brain function analogously to peripheral lymph vessels.^[^ ^5^ ^]^ Instead, the clearance of neurotoxic waste – such as protein aggregates or toxic metabolites – depends on cerebrospinal fluid (CSF) from the subarachnoid spaces that flow through arterial perivascular spaces, into the parenchyma to mix with interstitial fluid and solutes, and back into venule perivascular spaces to drain out of the brain. Solutes, antigens, and waste travel from these perivenous spaces to brain lymphatic vessels, specifically meningeal lymphatic vessels, which serve as the conduits through which these species are delivered to peripheral lymph nodes, specifically cervical lymph nodes. Perivascular spaces, also known as Virchow‐Robin spaces, are the periarterial (PAS), perivenous (PVS), and pericapillary spaces surrounding small arterioles, venules, and capillaries in the brain, respectively, created by the gap between the astrocytic endfeet, also known as the glia limitans, and the endothelium.^[^ ^6^ ^]^

**Figure 1:**
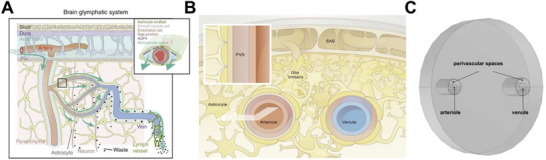
Anatomy of the glymphatic system. CSF flows along perivascular spaces created between brain vessels and the glial limitans of the blood‐brain barrier. A) Illustration of how CSF travels along penetrating arteries to collect waste in the brain and drain to lymph vessels surrounding veins. Adapted from Hablitz et al.^[^ ^72^ ^]^ B) Cross‐sectional view of a single arteriole‐venule unit, with associated glymphatic drainage system. Adapted from Asgari et al.^[^ ^31^ ^]^ C) Base anatomy of the arteriole‐venule unit in the COMSOL computational model.

CSF flow along these periarterial spaces and into the brain parenchyma is thought to be driven by differential hydrostatic pressure gradients generated by arterial pulsatility. When CSF enters the neuropil, it mixes with interstitial fluid and cellular waste. This composite enters perivenous spaces, becoming increasingly concentrated with metabolic by‐products as it effluxes to the meningeal lymphatic vessels in the dura mater – the outer layer of the meninges surrounding the brain – that in turn recirculate CSF and processes accumulating fluid and waste products.^[^ ^7^ ^]^ Aquaporin‐4 water channels embedded in the astrocytic endfeet surrounding the brain vasculature facilitate this directional flow through the brain parenchyma.^[^ ^8^ ^]^ The system is shown in two different perspectives in **Figure** **1**A,B.

Focused ultrasound (FUS) a therapeutic technology that entails delivering acoustic pressure waves to a small volume. One particularly powerful application of FUS is transient blood‐brain barrier opening (BBBO) for drug and gene delivery.^[^ ^9^, ^10^ ^]^ In this application of FUS, intravenously delivered microbubbles (MBs) undergo cycles of compression and rarefaction in the FUS field due to the inverse relationship between the pressure of the sinusoidal FUS wave and the MB volume.^[^ ^11^ ^]^ With proper FUS parameters, the expanding and contracting MBs exert mechanical stress on the surrounding vasculature that disrupts tight junctions and activates transcytosis.^[^ ^12^ ^]^ To date, numerous preclinical and clinical studies have actively investigated FUS BBBO as a mono‐ or combinatorial therapeutic technique.^[^ ^13^, ^14^, ^15^, ^16^, ^17^, ^18^, ^19^, ^20^ ^]^

Beyond allowing larger molecules to bypass the blood‐brain barrier, FUS BBBO produces a variety of changes in the underlying brain parenchyma, ranging from increasing the proportion of neuroprotective disease‐associated microglia in the brain's resident macrophage population (microglia) to improving nanoparticle transfection.^[^ ^21^, ^22^ ^]^ A recently identified additional effect is enhanced glymphatic waste clearance,^[^ ^23^ ^]^ including pathological proteins such as amyloid‐𝛽 in the setting of AD.^[^ ^24^, ^25^ ^]^ These studies suggest FUS treatment could positively impact diseases featuring glymphatic malfunction, such as Alzheimer's Disease and Parkinson's Disease.^[^ ^26^ ^]^

Despite the potential significance of FUS BBBO‐mediated clearance in the brain, the mechanisms through which this occurs are poorly understood. In fact, it was only recently that glymphatic features were identified in the human brain, and this feat was achieved only by using FUS BBBO or other novel technologies.^[^ ^27^, ^28^ ^]^ Previous computational models of the glymphatic system or brain vasculature have attempted to fill related knowledge gaps by simulating different aspects of glymphatic function, including perivascular pumping, the interface between perivascular spaces and the parenchyma, waste efflux routes, neuronal activity and vasomotion, and mass transport through the brain parenchyma.^[^ ^5^ ^]^ One notably modeled interstitial fluid flow in the mammalian brain parenchyma by estimating how idealized brain vasculature arrangements and orientations affect hydraulic resistance.^[^ ^29^ ^]^ Others have evaluated the possibility of advective mass transport as a waste clearance mechanism for glymphatics.^[^ ^30^, ^31^, ^32^ ^]^ Another explored how elevations in intracranial pressure change CSF dynamics in the brain.^[^ ^33^ ^]^ Still more have attempted to statistically predict the topology of brain vasculature or have tried to understand the role of glymphatics in the pathogenesis of neurodegenerative diseases like AD.^[^ ^34^, ^35^ ^]^ Nonetheless, to our knowledge, no computational modeling studies have simulated how FUS BBBO affects interstitial fluid flow and glymphatic clearance of different species. Herein, we leverage finite‐element modeling and COMSOL Multiphysics to understand how FUS BBBO enhances glymphatic waste clearance. We found that FUS BBBO promotes diffusion‐mediated mixing of brain solutes with CSF and increased CSF convection from arterioles to venules, which together promote faster clearance times.

## 2 Experimental Section

The model design was completed in COMSOL Multiphysics using the microfluidics package, which calculates time‐dependent pressure, velocity, and concentration values using the Brinkman equations and Fick's Law. All boundaries were defined as being open to both fluid and solute flux, with no‐slip boundary conditions on the model vessels. All parameter values were collected from the literature and are provided in **Table** **1**. FUS BBBO was modeled as changes in permeability (300% increase), porosity (167% increase), flow (0.8 µm s^−1^), and perivascular space geometries (175% increase), using the values provided in **Table** **2**. It should be noted that, in these simulations, FUS BBBO is not modeled as a delivery mechanism. Instead, FUS BBBO is treated as a system perturbation that affects the clearance of pre‐existing solutes in the brain parenchyma. FUS was modeled after the pre‐clinical FUS Instruments RK‐50 device. Solutes were modeled based on measured diffusion coefficients in the mammalian brain parenchyma ^[^ ^46^, ^53^, ^54^, ^55^ ^]^. The effects that FUS BBBO has on glymphatic waste clearance at different spatial targets, for different solutes, and for different solute distributions, were explored. Each experimental condition was tested in a unique simulation. All simulations were compared to each other, allowing us to observe the systemic changes with and without FUS BBBO for a tracer, amyloid‐β, and α‐synuclein. Constant temperature and pressure are assumed. It is also assumed that no chemical reactions are occurring and that any pressure contribution from the physical deformation of vessels caused by cardiac pulsations is accounted for in the pressure gradient between arteriole and venule. FUS BBBO is assumed to occur downstream of the vessels present in the model. Because of this, blood flow through the vessels and into the parenchyma via BBBO can be ignored. Finally, all tissue heterogeneity from microglia, astrocytes, neurons, or other brain tissue is captured by parameters like porosity and permeability. All models were simulated until 95% of the initial mass of the tracer was cleared, at which point the simulation ended. Temporal and spatial data related to solute flux, pressure, and flow were collected.

**Table 1:** Glymphatics model geometry and parameters.

Parameter	Value	Unit	Refs.
Length	100	um	[30, 34]
Arteriole Diameter	20	um	[36, 37, 38, 39]
Venule Diameter	30	um	[36]
Perivascular Space Width	10	um	[36, 37, 40]
Glia Limitans Width	1	um	[31, 36, 41]
Mean distance from arteriole to venule	280	um	[32, 33]
Parenchyma Porosity	20	%	[42, 43, 44]
Parenchyma Permeability	10^−12^	m^2^	[45]
Parenchyma Tortuosity	1.7	dimensionless	[46]
Glia Limitans Porosity	0.3	%	[45, 47]
Glia Limitans Permeability	5 × 10^−14^	m^2^	[32, 45]
Pressure Gradient Across Vessels	3	mmHg/m	[48, 49]
Periarterial CSF Velocity	18.7	um/s	[8, 40]
Perivenous CSF Velocity	3	um/s	[50]
CSF Viscosity	1	mPa/s	[51]
CSF Density	1	g/mL	[52]

**Table 2:** Parameters for modeling FUS and various solutes.

Parameter	Value	Unit	Refs.
**FUS Parameters**			
Focal Volume Cross‐Sectional Diameter	0.9	mm	[56]
Perivascular Space Size Increase	175	%	[57]
Parenchyma Porosity Increase	167	%	[58]
Parenchyma Permeability Increase	300	%	[59]
FUS Fluid Inlet	0.8	$\frac{\mathrm{um}}{s}$	[22]
**Solute Diffusion Coefficients**			
Soluble Tracer	10^−9^	$\frac{m^{2}}{s}$	[54, 55]
amyloid‐𝛽	1.8 × 10^−10^	$\frac{m^{2}}{s}$	[46]
ɑ‐synuclein	7.8 × 10^−10^	$\frac{m^{2}}{s}$	[53]

## 3 Results

### 3.1 Base Computational Model Design and Clearance of Centralized Tracer with FUS BBBO

**Figure 2:**
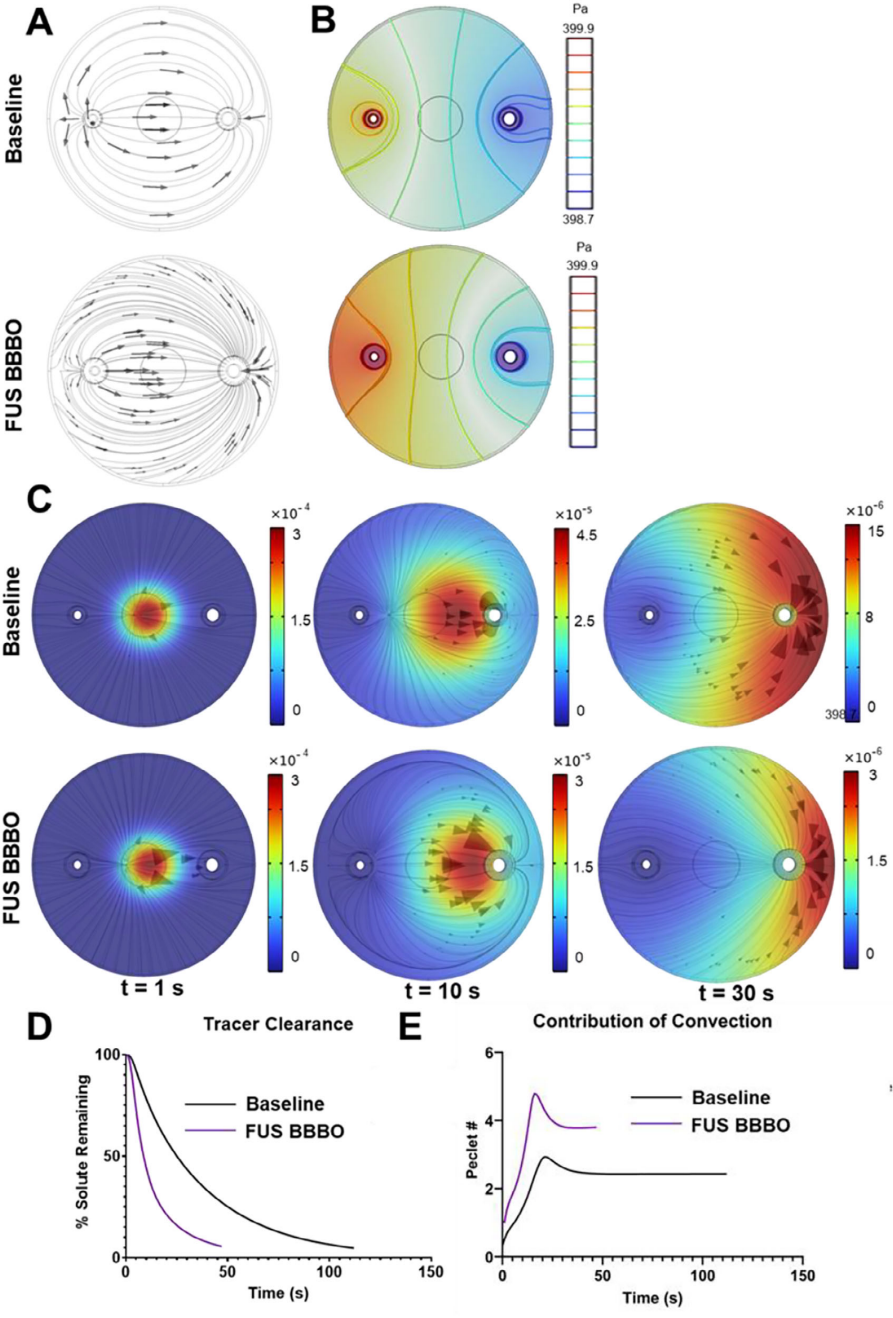
Simulation of fluid flow, pressure distribution, and tracer flux in the computational glymphatic transport model. A) CSF flow profile at baseline and after FUS BBBO. B) Pressure profile at baseline and after FUS BBBO. C) Species concentrations (color maps) and fluxes (arrows) at t = 1 s, 10 s, and 30 s respectively. Baseline and FUS BBBO conditions are represented. Color scale in units of $\frac{\mathrm{mol}}{m^{3}}$. D) Solute remaining over time for both the baseline FUS BBBO models. E) Comparison of the Péclet number (ratio of convection to diffusion) for the baseline and FUS BBBO models.

To simulate the effects of FUS BBBO on the vasculature of the mammalian brain, we first used in vivo literature values to construct a microscopic baseline model of a single arteriole‐venule (AV) neurovascular unit. Figure 1C shows the base model's design and outputs next to an illustration of the in‐vivo anatomy of the system being modeled (Figure 1B). The single AV unit was modeled to characterize CSF ingress and egress dynamics in the glymphatic system. Initially, we investigated the clearance dynamics of a macromolecule modeled as a cylindrical bolus of a tracer centralized between the vessels. To model FUS BBBO, both perivascular spaces were enlarged by 175% as previously reported and fluid inlets were added around the perimeter to simulate fluid influx.^[^ ^22^, ^57^ ^]^ The changes in the CSF flow profile before and after FUS BBBO are shown in **Figure** **2**A. Similarly, FUS BBBO produces effects on the fundamental convection‐driving pressure gradient (Figure 2B). The simulated tracer was modeled with a diffusion coefficient (DC) of $10^{-9}\frac{m^{2}}{s}$, analogous to a macromolecule.^[^ ^55^ ^]^ The small circular shape of the tracer at t = 0 was chosen because it best visually illustrates subsequent fluid flow in the model. In the FUS BBBO model, we see an accelerated clearance of the tracer into the perivenous spaces compared to baseline, with the FUS BBBO model reaching the 95% clearance criterion in 46.5% of the time of the base model (Figure 2C,D). This difference is visualized in Figure 2D, which shows the percentage of remaining solute is calculated as a function of time for both baseline and FUS BBBO conditions. Our models indicate enhanced tracer clearance (Figure 2D) and increased convection relative to diffusion for FUS BBBO relative to baseline (Figure 2E), suggesting that FUS BBBO‐augmented convection drives faster clearance.

### 3.2 Distributed Tracer Model

**Figure 3:**
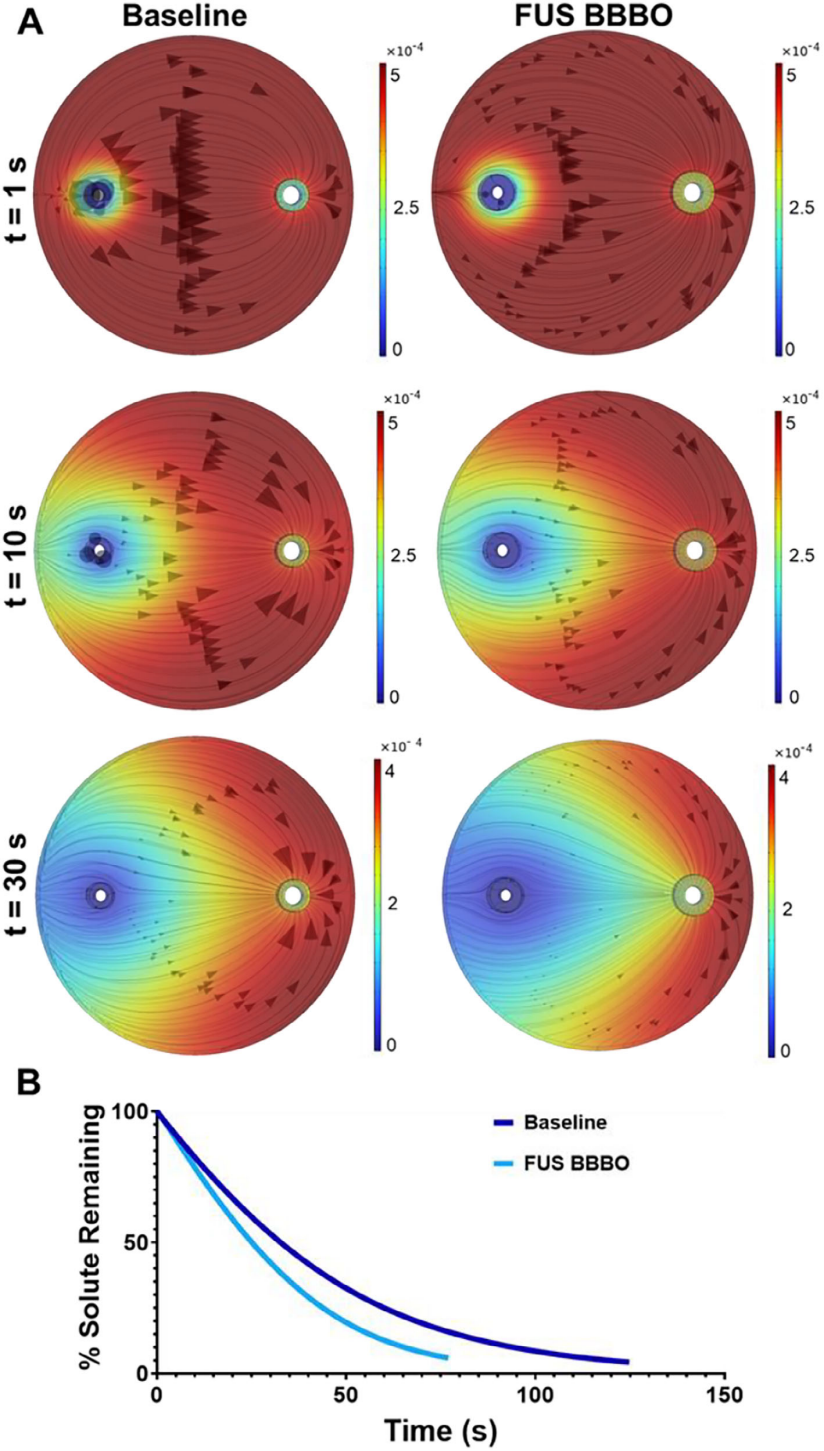
Simulation of clearance of a tracer that is uniformly distributed throughout the parenchyma at time t = 0. A) Species concentrations (color maps) and fluxes (arrows) at t = 1 s, 10 s, and 30 s. Baseline (A) and FUS BBBO conditions (B) are simulated. Color scale in units of $\frac{\mathrm{mol}}{m^{3}}$. B) Tracer clearance as a function of time for baseline and FUS BBBO simulations.

We next simulated the scenario wherein the tracer was distributed evenly throughout the brain parenchyma at t = 0. In doing so, the tracer more accurately represents how homogeneously distributed waste would be expected to be cleared via the glymphatic system. Because the flow profile and pressure gradient are unaffected by the solute distribution, the baseline distributed tracer model predicts that the basic movement of tracer from the PAS, across the parenchyma, into the PVS, and then out of the system, is unaffected by solute distribution (**Figure** **3**A). Similar to the bolus tracer scenario, we again see that FUS BBBO accelerates solute clearance of the homogeneously localized tracer, with particularly elevated removal of the solute near the PAS (Figure 3B,C). Here, solute clearance is clearly accelerated with FUS BBBO, which yielded a ≈33% reduction in time to reach the 95% tracer clearance criterion in the model when compared to baseline. The Péclet number increased 160% in the presence of FUS BBBO. We also ran analyses of the distributed tracer model's sensitivity to changes in perivascular space radius, permeability, and porosity upon application of the FUS BBBO stimulus (Figure S1, Supporting Information). The model behaved as expected in all cases. Increasing perivascular radius yielded modest increases in solute clearance, while reducing this value by 50% markedly reduced clearance, highlighting the importance of this physical factor (Figure S1A, Supporting Information). Reducing permeability by an order of magnitude to 1E‐13 m^2^ also caused a sharp drop in clearance (Figure S1B, Supporting Information). Increasing porosity led to reduced glymphatic clearance (Figure S1C, Supporting Information) because, given the constant flow input following FUS BBBO, fluid velocity decreases in the pores, thereby reducing advection as well.

### 3.3 Amyloid‐β and ɑ‐Synuclein Clearance

**Figure 4:**
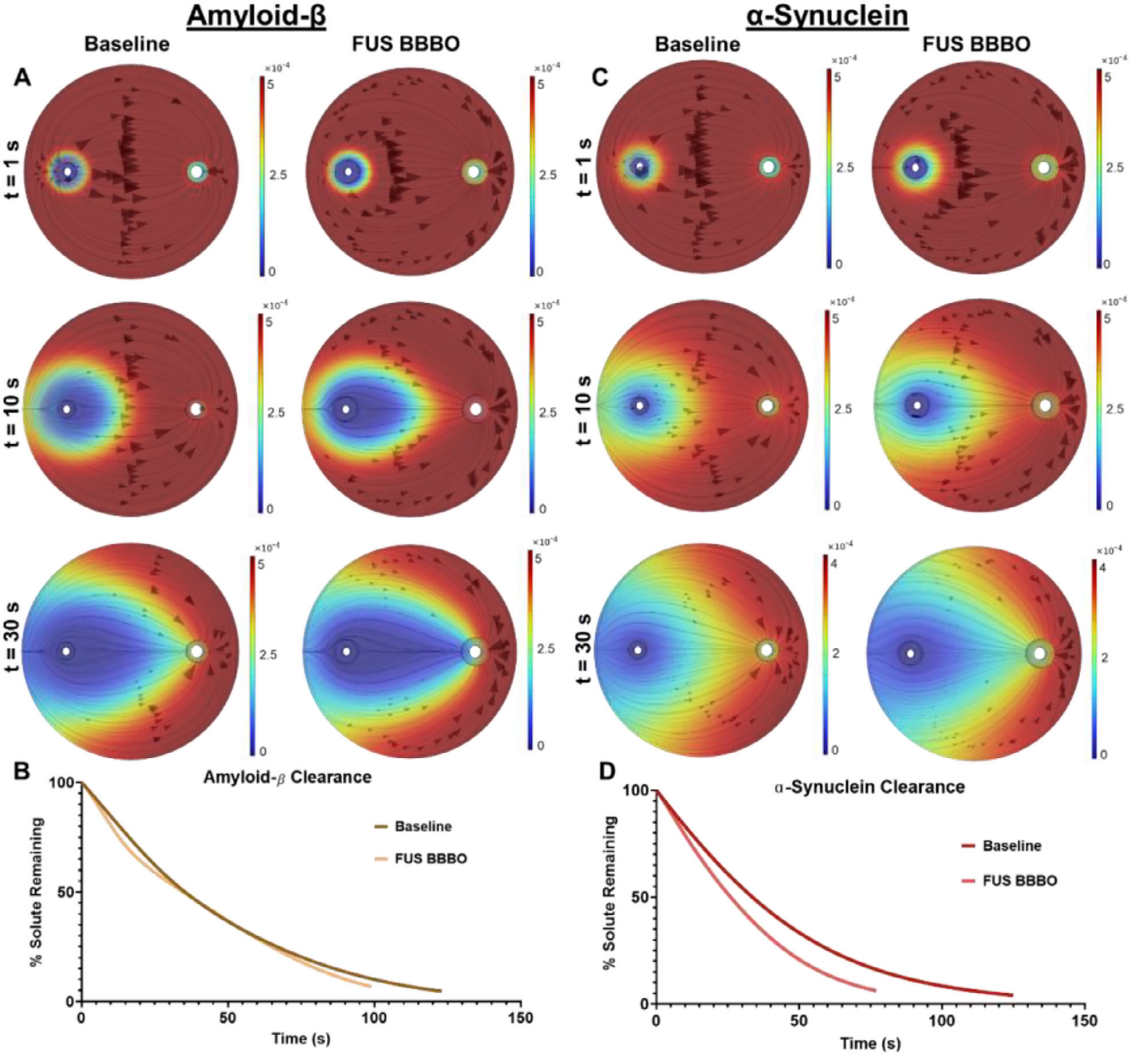
Simulation of clearance of amyloid‐β and α‐synuclein when the species are uniformly distributed throughout the parenchyma at time t = 0. A. Amyloid‐β concentration (color map) and flux (arrows) at t = 1 s, 10 s, and 30 s, for both baseline and FUS BBBO conditions. Color scale in units of $\frac{\mathrm{mol}}{m^{3}}$. B) Amyloid‐β clearance as a function of time for baseline and FUS BBBO simulations. C) Alpha‐Synuclein concentration (color map) and flux (arrows) at t = 1 s, 10 s, and 30 s, for both baseline and FUS BBBO conditions. Color scale in units of $\frac{\mathrm{mol}}{m^{3}}$. D) Alpha‐Synuclein clearance as a function of time for baseline and FUS BBBO simulations.

The accumulation of amyloid‐β and α‐synuclein are thought to contribute to the pathological progression of AD and Parkinson's Disease, respectively.^[^ ^60^, ^61^ ^]^ It has been reported that FUS BBBO can accelerate glymphatic clearance of amyloid‐β.^[^ ^62^ ^]^ Because it is hypothesized that glymphatic malfunction plays a crucial role in removing many other neurotoxic proteins from the brain, similar mechanisms likely exist for species like α‐synuclein despite not yet being characterized.^[^ ^63^, ^64^ ^]^ To predict how FUS BBBO augments the glymphatic clearance of these species, we changed the DC of the tracer to that for soluble amyloid‐𝛽 ($1.8\times 10^{-10}\frac{m^{2}}{s}$) and ɑ‐synuclein ($7.8\times 10^{-10}\frac{m^{2}}{s}$). First, we modeled amyloid‐β concentration flux through time for baseline and FUS BBBO conditions (**Figure** **4**A,B). Here, the model unexpectedly predicts that FUS BBBO has only a minimal influence on amyloid‐β clearance, with FUS BBBO reducing the time to 95% clearance by only ≈12% (Figure 4C). Next, we modeled α‐synuclein concentration fluxes through time for baseline and FUS BBBO conditions (Figure 4D,E), respectively. For this protein, the model predicts that FUS BBBO will accelerate clearance by ≈33% (Figure 4F). Clearance times for the “centralized tracer” (Figure 2), “distributed tracer” (Figure 3), and amyloid‐β and α‐synuclein (Figure 4) are compared in Figure S2 (Supporting Information). This data suggests that FUS BBBO produces differential effects based on the distribution and diffusivity of proteins in the brain.

### 3.4 Solute Model Flux Comparisons

**Figure 5:**
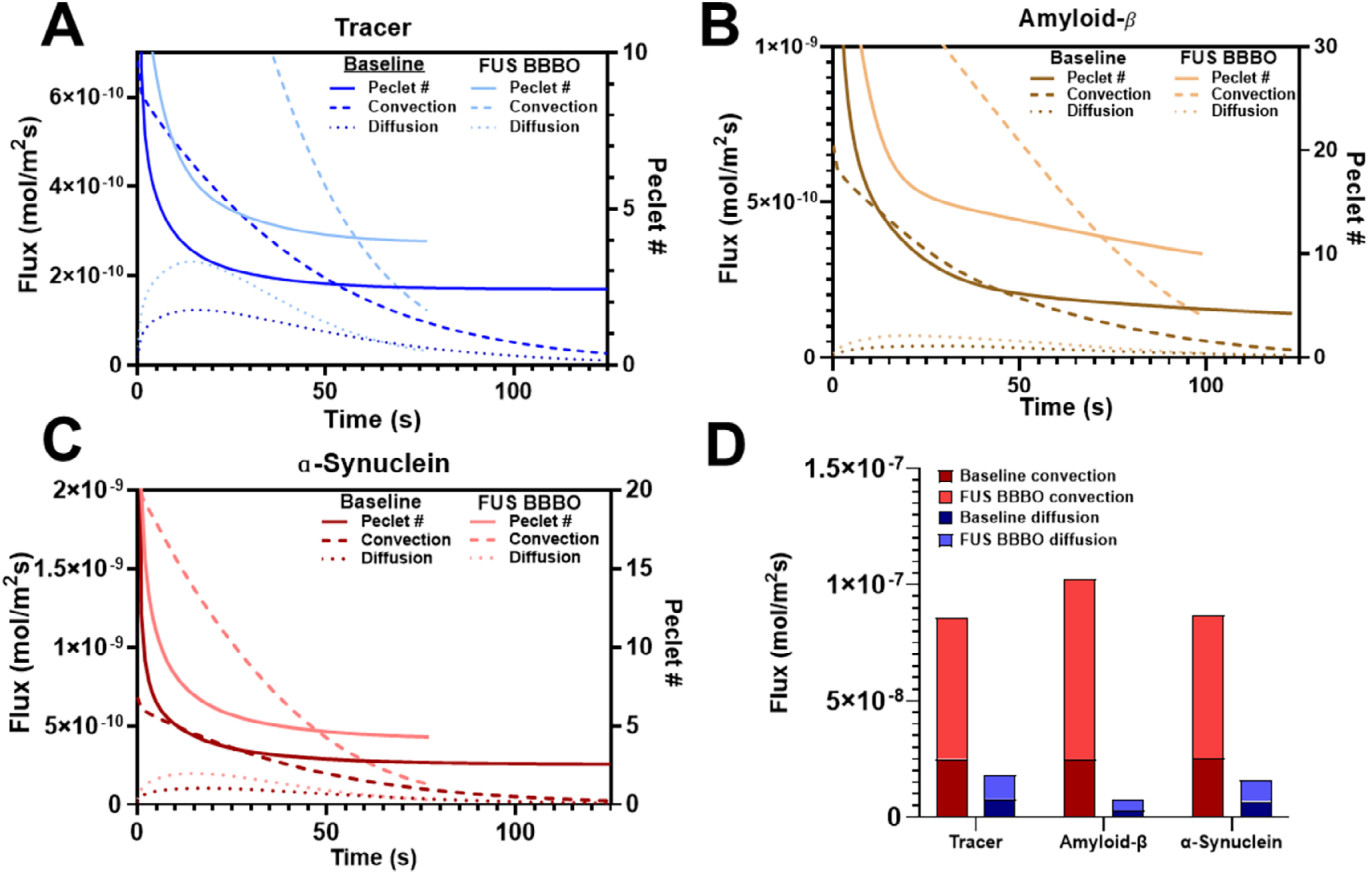
Line graphs of flux dynamics and Péclet number as a function of time for both baseline and FUS BBBO conditions. A) Tracer simulations. B) Amyloid‐β simulations. C) Alpha‐synuclein simulations. D) Summary of FUS BBBO‐mediated changes in flux dynamics for tracer, amyloid‐β, and α‐synuclein.

We then asked how convection and diffusion were affected through time by FUS BBBO. To this end, convective and diffusive flux over time, are shown alongside the Péclet number in **Figure** **5** for the tracer (Figure 5A), amyloid‐β (Figure 5B), and α‐synuclein (Figure 5C) to highlight the specific effects of FUS BBBO for each species. Figure 5D shows the cumulative change in magnitude of these fluxes as well. Note that, for all 3 species being simulated, both convection and diffusion increase. However, as reflected by the Péclet number, which is a representation of the relative contribution of convective to diffusive flux that also increases for all 3 species, FUS BBBO consistently enhances convective flux more than diffusive flux. Interestingly, despite FUS BBBO predicting a smaller acceleration of amyloid‐β clearance (Figure 4C), FUS BBBO exerts the greatest impact on Péclet number in the amyloid‐β simulation (250% increase) (Figure 5B) when compared to the tracer (160% increase) (Figure 5A) and α‐synuclein (200% increase) (Figure 5C). To illustrate spatial differences in Péclet number amongst the 3 different simulations, both at baseline and after FUS BBBO, a heat map of the Péclet number is shown in Figure S3 (Supporting Information). These data indicate that the effect that FUS BBBO has on different solutes is a result of the nuanced interplay of these modes of mass transport.

### 3.5 Glymphatic Clearance at the Edge of the Focus

**Figure 6:**
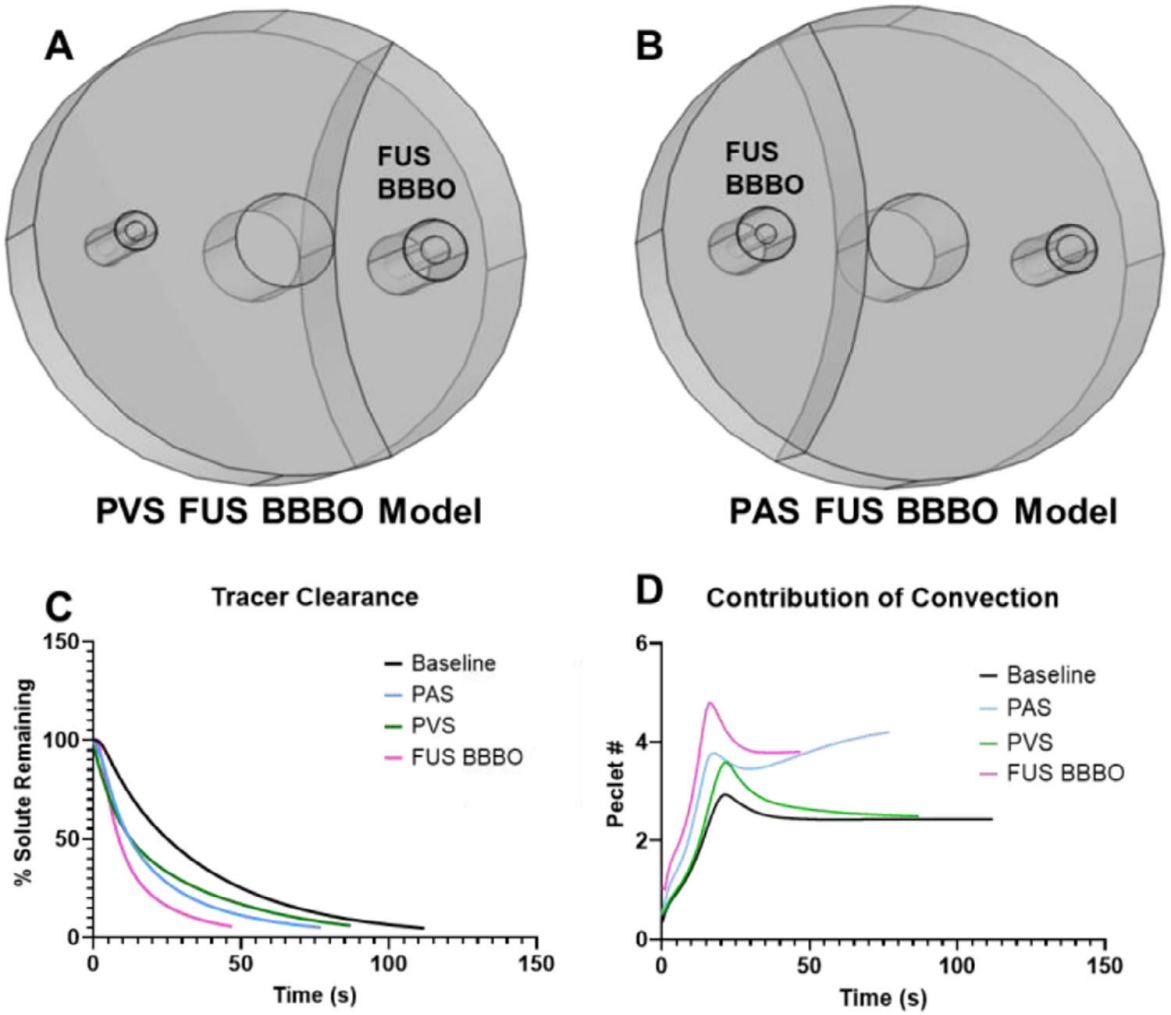
Simulations of glymphatic transport when the edge of the focus, shown as an arc in the images, lies between the arteriole and venule. A) Illustration of the scenario wherein FUS BBBO only occurs on the venous side of the model, denoted as “PVS FUS BBBO Model”. B) Illustration of the scenario wherein FUS BBBO only occurs on the arterial side of the model, denoted as “PAS FUS BBBO Model”. C,D) Tracer clearance (C) and Peclet number (D) for PVS FUS BBBO and PAS FUS BBBO simulations in comparison to baseline and FUS BBBO simulations. For FUS BBBO, the entire model is exposed to FUS BBBO.

For any given FUS BBBO treatment, arteriole‐venule units at the edge of the focus will only receive partial treatment. In other words, the edge of the focus will intersect the unit, such that part of the unit is exposed to FUS BBBO, while the remainder is not. Because of this, we tested (i) how partial FUS BBBO treatment affects overall glymphatic clearance and (ii) how FUS BBBO applied only on the arterial side compares to FUS BBBO applied only on the venous side. To this end, we simulated conditions wherein only a fraction of the modeled volume was exposed to FUS BBBO. These simulations are denoted as “PVS FUS” (**Figure** **6**A) and “PAS FUS” (Figure 6B), with the focus covering only the venous or arterial side of the model, respectively. For these simulations, the tracer was placed in the centralized location at time t = 0, as in Figure 2. We then calculated the tracer clearance and Péclet number for the PVS FUS and PAS FUS simulations, as well as for the baseline and FUS BBBO simulations wherein the tracer is evenly distributed throughout the parenchyma at time t = 0 and the focus covers the entire model (Figure 6C,D). Comparing partial FUS coverage (i.e., PVS FUS and PAS FUS) to full FUS coverage (i.e., whole) demonstrates a greater increase in clearance with full FUS coverage, which presumably generates a greater stimulus for transport (Figure 6C). Interestingly, the “PAS FUS” model cleared faster and experienced a higher Péclet number than the “PVS FUS” model, despite the exact same FUS focal volume for both models. These models indicate that different FUS targeting regions could produce different effects depending on the selectivity of the focal region.

## 4 Discussion

FUS BBBO is now widely deployed for targeted drug and gene delivery to the brain, in both pre‐clinical and clinical settings.^[^ ^65^ ^]^ Further, a potentially beneficial secondary effect of FUS BBBO is the augmentation of glymphatic clearance, which also has therapeutic potential.^[^ ^23^ ^]^ However, the biotransport mechanisms underlying this glymphatic augmentation effect of FUS BBBO are poorly understood. To address this knowledge gap, we developed and implemented a COMSOL Multiphysics model of an arteriole‐venule pair in the brain, with associated parenchyma. The model predicts that FUS BBBO shortens the time needed for mass to clear tissue and increases the Péclet number. FUS BBBO promotes the movement of CSF and solute from the arteriole to the venule, but flux is largely dependent on the implicit interactions between the solute and the medium through which it is moving. Solutes more centralized to the CSF flow profile and that have a higher diffusion coefficient tend to clear more rapidly due to increased convection and enhanced diffusive mixing. The magnitude of FUS BBBO effects corresponds to the size of the focal volume, with slightly greater effects for sonicated arterioles compared to sonicated venules. The model can predict the impact of FUS BBBO on the clearance of different species, which can both inform therapeutic strategies and elucidate mechanisms of secondary responses to FUS BBBO.

### 4.1 Influence of Initial Tracer Distribution

The centralized tracer model (Figure 2) cleared in 46.5% of the amount of time with FUS BBBO as compared to without, while the distributed model (Figure 3) cleared in 67% of the base time when FUS was present. While this might suggest that FUS BBBO preferentially enhances the clearance of aggregated solute over dispersed solute, the initial concentration of tracer is held constant between models, not the mass. Hence, the homogenous distribution models have an overall larger initial mass. Regardless, this should not affect the magnitude of the FUS BBBO effects, especially because the rate of solute clearance decreases with less solute in a manner reminiscent of an exponential decay function. An explanation for why FUS BBBO might better enhance aggregated solutes is that, by greatly improving convection, more CSF flows directly down the pressure gradient, between the arteriole and venule, compared to peripheral paths. In the centralized models, this would lead to more rapid clearance, while in the distributed models, the solute on the periphery would take longer to clear. Overall, the models suggest that FUS BBBO can shorten waste clearance to almost half the time in the worst case for various solutes. This finding agrees with other studies and adds to them by suggesting that aggregated, but still soluble, solutes located directly between vessels might be the most affected by FUS BBBO‐enhanced glymphatic clearance.^[^ ^23^, ^24^ ^]^

### 4.2 Influence of Different Solutes

The solute characteristics were determined by each species’ measured DC in the mammalian brain parenchyma. The tracer had the highest DC, followed by ɑ‐synuclein, and finally by amyloid‐ β. This corresponds to FUS‐BBBO mediated increases in Péclet number of 160%, 200%, and 250%, respectively. It also trends with FUS‐BBBO mediated decreases in time to clear solute of 33.3%, 33.7%, and 12.4%, respectively. These results challenge the notion that convection is the sole driver in FUS BBBO‐enhanced solute clearance and instead suggests that the DC and diffusive flux play major roles in glymphatic waste clearance. Why would amyloid‐𝛽, with the largest proportional increase in convection, exhibit the smallest decrease in clearance time? We believe the answer lies in Figure 5 and Figure S2 (Supporting Information), which illustrate the relationship between the ability of a solute's propensity for diffusion and convection. Diffusion coefficients are determined experimentally by measuring the amount of flux of a solute in a certain medium with a certain concentration gradient, essentially indicating how well a solute can diffuse in a medium. One might initially consider convection and diffusion opposing processes, and indeed in many systems they can be. Convection can create concentration gradients that increase in the same direction as flux, whereas diffusive flux always occurs down a concentration gradient. However, for a solute to advect, a certain degree of mixing is required between the flowing solvent and the solute. This mixing process is mediated by diffusion, meaning that species that diffuse more efficiently will also mix with a convecting fluid more efficiently. This explains why the tracer and ɑ‐synuclein experienced a more pronounced clearance enhancement with FUS BBBO. With larger DCs, they are better at diffusive mixing with the CSF and thus advect quickly out of the model. As can be seen in Figure 5, these species demonstrated a notably larger FUS BBBO‐mediated increase in peak diffusive flux compared with amyloid‐ β.

**Figure 7:**
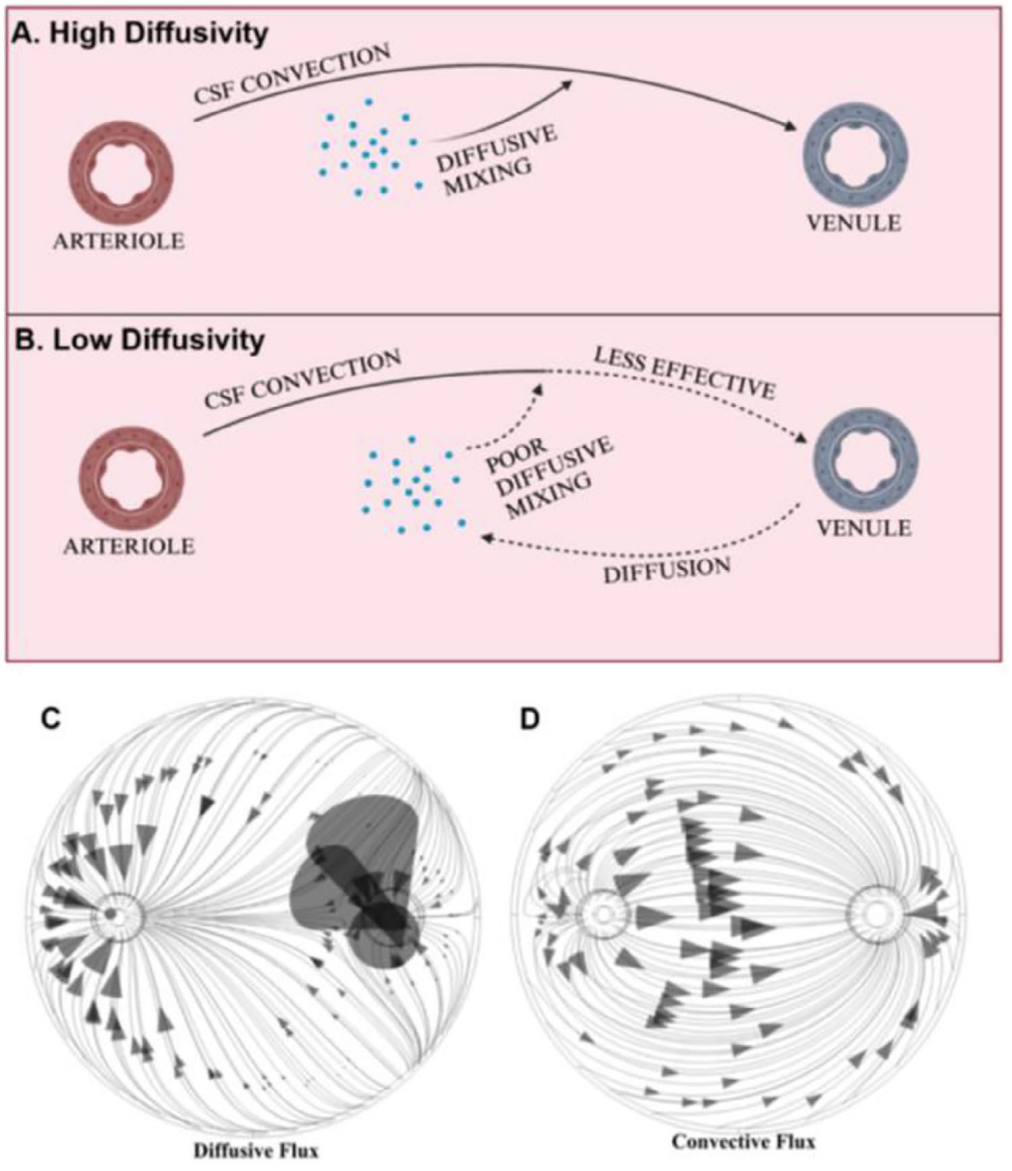
Influence of diffusion‐mediated mixing on glymphatic transport. A) Convection is enhanced by diffusion‐mediated mixing of the solute with CSF. B) Species with low diffusivity mix poorly with CSF, which hinders convection and increases diffusive flux. C) Diffusive flux profile D) Convective flux profile.

Additional questions are why amyloid‐𝛽 has a larger increase in Péclet number and why does Figure 5 indicate that amyloid‐𝛽 experienced more enhanced convective (≈4x increase compared to ≈3x) and diffusive (1.6x increase compared to ≈1.3x) flux than the other species? With a lower DC, amyloid‐𝛽 mixes poorly with CSF and convects less efficiently. This increases the simulation duration, which provides amyloid‐𝛽 with more time to diffuse. Because glymphatic flow concentrates solute around the venule, diffusion will occur away from the perivenular space, opposing convection. This will increase the overall amount of diffusive flux. The model will continue to run until the CSF eventually carries that solute toward the venule, which will increase the overall amount of convective mass flux as well. This back‐and‐forth process of opposing convection and diffusion, which is illustrated in **Figure** **7** and is promoted by poor mixing, increases the overall amount of flux that occurs in the simulation without improving the clearance efficiency. Figure 7 also shows an example of how the convection and diffusion flux profiles of a solute oppose one another for the FUS BBBO tracer model. Near the venule, diffusive flux points into the PVS because the CSF flowing therein acts as a diffusive sink. Figure S3 (Supporting Information) shows how amyloid beta less efficiently utilizes this CSF sink. Looking at the Péclet number for amyloid‐ β in Figure S3 (Supporting Information), diffusion plays a larger role near the arteriole than it does for other solutes, illustrating that this less‐diffusive species successfully diffuses against the concentration gradient generated by convection more so than the other species. Interestingly, there is less diffusion around the venule and less convection inside the perivenous space, indicating that amyloid beta fails to diffusively mix and travel with CSF. Overall, these data suggest that CSF‐mixing is an essential part of glymphatic waste clearance, and that FUS BBBO increases transport of CSF without improving this mixing process.

This insight also suggests that FUS BBBO will best enhance peak diffusion for solutes with a higher DC. This will improve mixing and minimize diffusive flux, thereby enhancing glymphatic clearance. A higher peak diffusion corresponds to better CSF mixing and not just diffusion toward a CSF “sink” in the perivenule space because studies have shown that an agent like Albumin (DC = 1.6E‐11 *m* ^2^/*s*) would take over 100 h to diffuse through 1 cm of brain tissue, which is not efficient enough to maintain human brain homeostasis.^[^ ^26^ ^]^

We believe our simulations offer insights into 2 important unresolved questions. First, they may inform our understanding of the relative contributions of advection and diffusion to solute flux through neuropil. Indeed, as reviewed by Bohr et al.^[^ ^66^ ^]^ there is considerable experimental evidence both for and against advection‐dominated transport. As presented in Figure 5D, our model predicts that both advection and diffusion are present, with advection dominating for all species under consideration. However, as noted previously, our model also predicts that diffusion is critical for bulk solute flux via mixing, a nuance that may be difficult to observe experimentally. Of course, going forward, as more empirically‐derived information about transport in neuropil is acquired, the model can be adjusted accordingly.^[^ ^22^, ^30^, ^31^, ^32^ ^]^Second, our findings also suggest that less diffusive agents, like amyloid‐ β, might not depend on the glymphatic system as the primary mode of clearance. FUS BBBO only enhanced clearance of amyloid beta by ≈10%, indicating that, even with a technology that augments this waste clearance system, solutes with low diffusivity likely depend on another mechanism to be cleared, such as enhanced microglial activation and phagocytosis.^[^ ^25^, ^67^, ^68^ ^]^ This notion is supported by the idea that a slower glymphatic clearance time increases the opportunities for microglia to surveil and phagocytose solutes, and by studies that have shown that amyloid beta and microglia colocalize in AD models.^[^ ^68^, ^69^, ^70^ ^]^ Indeed, >50% reductions in amyloid‐β plaque load have been attributed to microglial phagocytosis after ultrasonic microbubble activation in APP23 mice.^[^ ^68^ ^]^ Intriguingly, microglia consistently aggregate with amyloid‐ β in perivascular spaces, which further suggests that microglia might compensate for solute‐specific inefficiencies of the glymphatic system in disease contexts.^[^ ^69^ ^]^ Likely, many pathological solutes depend on a combination of clearance mechanisms, including the glymphatics system, to varying degrees. This finding can shape the optimization of therapeutic strategies for various neurodegenerative diseases and adds to our understanding of brain homeostasis.^[^ ^21^ ^]^Perhaps optimizing these effects, instead of glymphatic clearance‐enhancing effects, will produce a favorable therapeutic outcome.

### 4.3 Glymphatic Clearance at the Edge of the Focus

For simulations wherein the edge of the focus was placed between the arteriole and venule (Figure 6), the “PAS FUS BBBO” model cleared faster than the “PVS FUS BBBO” model, despite the exact same FUS focal volume for both models. This outcome could be related to the unique Péclet number seen in the “PAS FUS BBBO” model, which doesn't stabilize at a certain value like the other models but instead continues increasing rapidly up until the simulation terminates. A possible explanation could be that the FUS BBBO‐induced increase in perivascular space volume (≈175%) had a larger effect on the periarterial side because this side is where CSF enters the neuropil, despite the PVS having an overall larger volume increase due to a larger initial volume. A larger PAS could mean more influx of CSF and more convection, whereas an increased PVS might simply increase the rate at which CSF flows through the parenchyma. In other words, perhaps the rate of CSF the PAS allows through the vessels into the parenchyma acts as a bottleneck upstream of the PVS, as the PAS determines the rate of CSF flux that the PVS in turn then accommodates. An alternative explanation could be that FUS influences the pressure gradient that drives glymphatic CSF flow. On the PAS side, additional fluid inlets and an increase in pressure could increase the rate of waste clearance. On the PVS side, it would follow that more fluid inlets and increased pressure would oppose, and thus less efficiently enhance, glymphatic flow.

### 4.4 Model Novelty, Limitations, and Opportunities

Numerous computational models of glymphatic function have been developed, but we believe ours in the first to provide spatiotemporal maps of 3D pressure, velocity, and concentration flux profiles in the presence of FUS BBBO. Some models individually explore pressure, concentration flux over time, 2D models of spatial convection, or flow velocities, but none integrate and visualize these data in a 3D format.^[^ ^32^, ^41^, ^48^, ^71^ ^]^ Furthermore, there are currently no models that explore the effects of FUS BBBO on the glymphatics system. Our current study extends the field by incorporating 3D pressure, velocity, and concentration flux profiles into this model. Therefore, this model provides unique insights and can be further leveraged in the future for computational studies aimed at understanding glymphatic flow in healthy and pathological settings.

This model does have limitations. The model has a simple geometry in that no vessels are perfectly cylindrical or parallel. Thus, it does not account for systemic effects of FUS BBBO on the glymphatics. Another potential limitation is that perivascular spaces are notably elliptical, not annular as modeled herein.^[^ ^36^, ^40^, ^72^ ^]^ Additionally, the brain parenchyma is characterized by only a few parameters. There is anisotropy along neuronal tracts, while this model assumes isotropy throughout the parenchyma.^[^ ^73^ ^]^ Further, the DC of various solutes through brain microstructures, like the one micron‐thick glia limitans, has not been measured in vivo. Therefore, only the DCs of the solutes in the parenchyma were used. We also acknowledge that it was necessary to collect parameters from different mammalian species, the directionality of FUS energy was not considered, and the temporal dynamics of BBB recovery after FUS BBBO was not implemented. That said, we submit the assumptions that are made are realistic and do allow the investigation of the glymphatics system and the bioeffects of FUS BBBO. The insights produced by this study might otherwise have been difficult or impossible to produce.

Finally, while FUS BBBO was the only stimulus in our simulations, recent studies report that glymphatic clearance is also augmented in response to FUS in the absence of MBs.^[^ ^74^, ^75^, ^76^ ^]^ Because opening of the BBB with FUS at low‐intensities in the absence of intravascular MBs is highly unlikely, these interventions are likely augmenting glymphatic clearance via modulation of cell signaling pathways that subsequently affect physical tissue properties, such as perivascular space dimesnions and tissue permeability. Going forward, should sufficient quantitative information become available, we submit the model could be a valuable tool for predicting how tissue modulation in response to FUS would be expected to impact glymphatic clearance.

## Conflict of Interest

The authors declare no conflicts of interest.

## Supporting information



Supporting Information

## Data Availability

The data that support the findings of this study are available from the corresponding author upon reasonable request.
