# Supplementary material for: Computational Fluid Dynamic Model Prediction of Enhanced Glymphatic Clearance in Response to Focused Ultrasound‐Mediated Blood‐Brain Barrier Opening
Source: Adv Sci (Weinh). 2025 Sep 4;12(44):e10684. doi: 10.1002/advs.202510684 (PMC12667546; doi:10.1002/advs.202510684)
Supplement: Supplementary file 1 — Supporting Information [file ADVS-12-e10684-s001.docx]

Supporting Information

Computational Fluid Dynamic Model Prediction of Enhanced Glymphatic Clearance in Response to Focused Ultrasound-Mediated Blood-Brain Barrier Opening

Ryan A. Gladwell, Delaney G. Fisher, Joshua D. Wythe, Christopher B. Highley, John R. Lukens, and Richard J. Price*

**
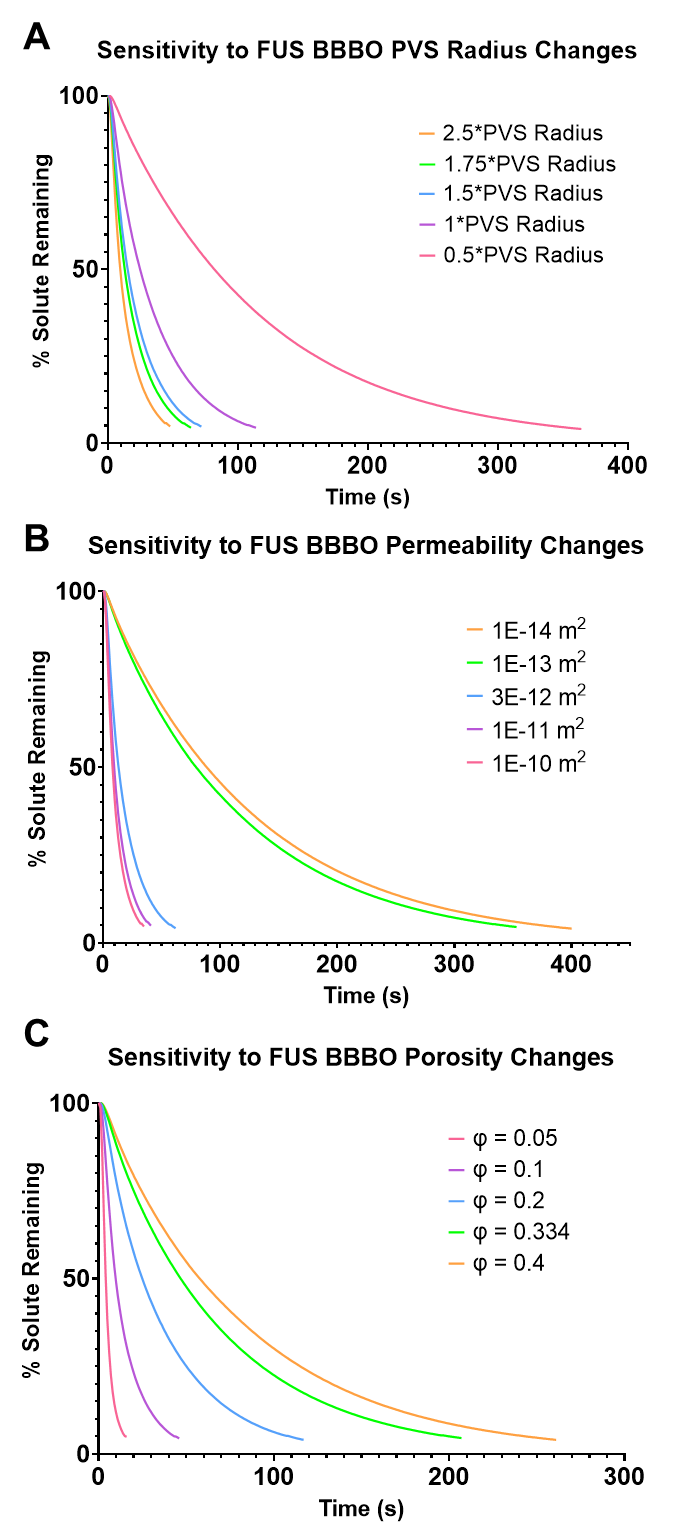
Figure S1. Sensitivity of the model to FUS BBBO-induced changes in perivascular space, permeability, and porosity. A)** Sensitivity of solute clearance to perivascular space size. **B)** Sensitivity of solute clearance to permeability. **C)** Sensitivity of solute clearance to porosity changes.


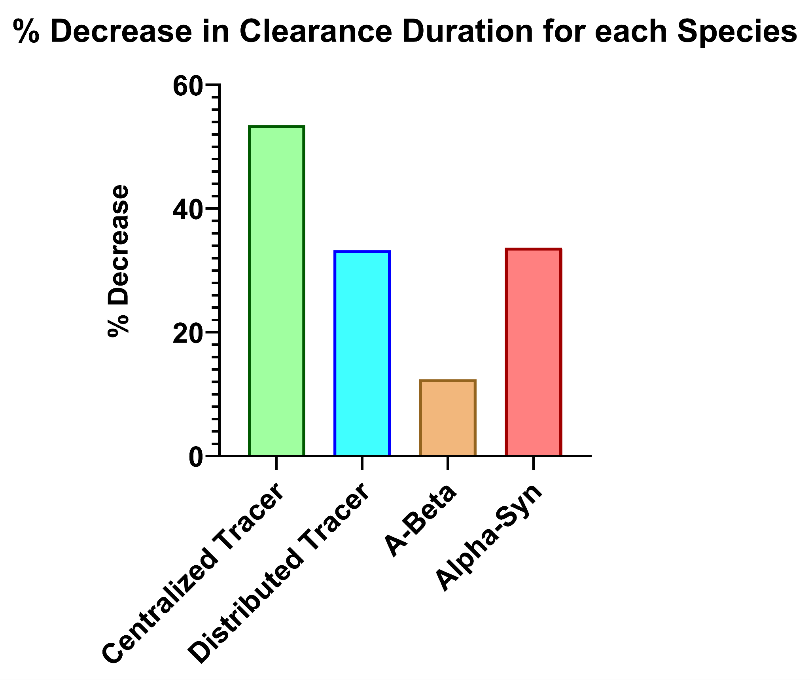
**Figure S2.** FUS BBBO-mediated decrease in clearance time for each tested species.

**
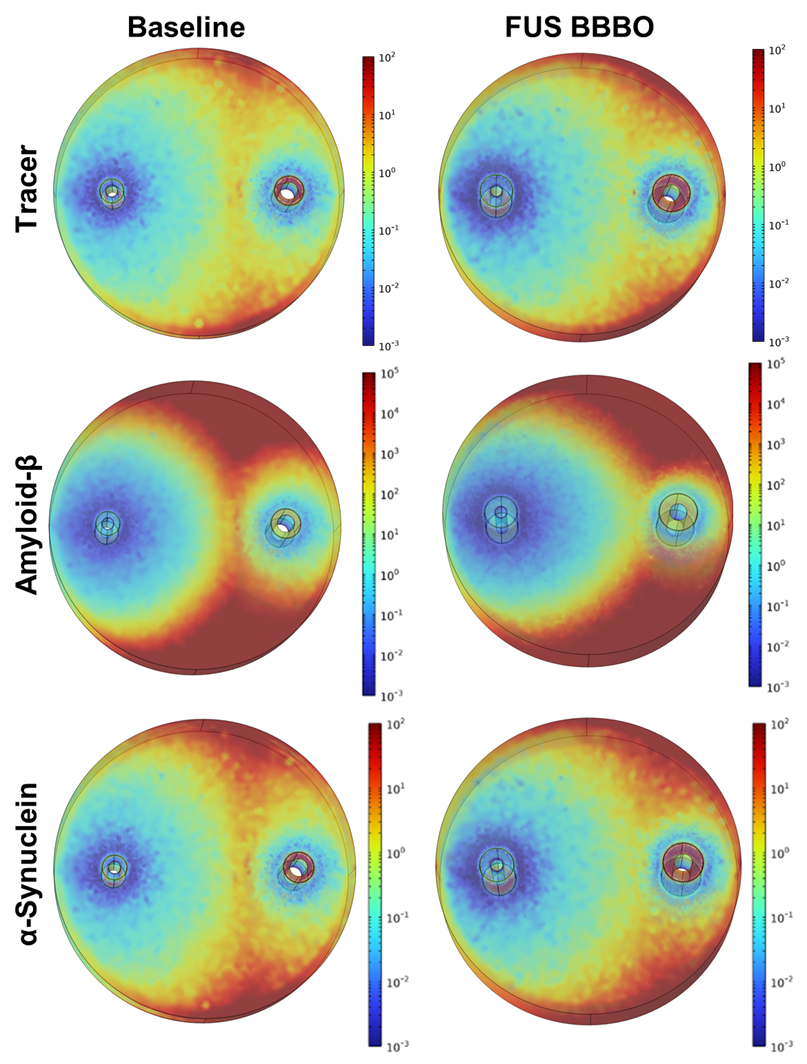
Figure S3. Peclet number distributions for tracer, amyloid-β, and α-synuclein simulations, both at baseline and 5 s after FUS BBBO.** The Péclet number is dimensionless because it is a ratio of convective flux to diffusive flux. Thus, PN > 1 indicates convective dominance, while PN < 1 indicates diffusive dominance. Axes are logarithmic to emphasize the prevailing flux patterns. Image taken at t = 5 s.
